# Improvements in Hemodynamics and Right Heart Remodeling Following Balloon Pulmonary Angioplasty Treatment in Patients With Chronic Thromboembolic Pulmonary Hypertension: A Retrospective Study

**DOI:** 10.1002/hsr2.70159

**Published:** 2024-11-13

**Authors:** Qi‐Le Shen, Qin‐Hua Zhao, Hui‐Ting Li, Jie Deng, Jing He, Lan Wang, Su‐Gang Gong, Jin‐Ming Liu

**Affiliations:** ^1^ School of Medicine Tongji University Shanghai China; ^2^ Department of Emergency, Shanghai Tongren Hospital Shanghai Jiaotong University School of Medicine Shanghai China; ^3^ Department of Pulmonary Circulation Shanghai Pulmonary Hospital, Tongji University School of Medicine Shanghai China; ^4^ Dermatology Hospital Southern Medical University Guangzhou China

**Keywords:** balloon pulmonary angioplasty, chronic thromboembolic pulmonary hypertension, echocardiography, right cardiac catheterization

## Abstract

**Background and Aims:**

This study aimed to evaluate the hemodynamic alterations and right heart remodeling dynamics in patients with inoperable chronic thromboembolic pulmonary hypertension (CTEPH) undergoing treatment with balloon pulmonary angioplasty (BPA).

**Methods:**

This retrospective cohort study involved a cohort of 31 patients with a confirmed diagnosis of CTEPH. Comprehensive clinical evaluations were systematically performed before BPA, and at 3 and 6 months following the procedure.

**Results:**

Significant clinical progress was evidenced by the uplift in the percentage of patients achieving WHO‐FC II, escalating from 19.35% at baseline to 51.61% at 6 months after BPA (*p* = 0.003). NT‐proBNP levels significantly dropped from a median of 614.6 to 69.9 pg/mL (*p* < 0.001). Hemodynamic assessments showed significant decreases in mean PAP from 45.53 ± 11.19 to 22.56 ± 5.81 mmHg (*p* < 0.001) and PVR from 8.33 to 2.86 WU (*p* < 0.001). Echocardiographic analysis revealed substantial reductions in the right atrial area (RAA, from 20.29 ± 7.55 to 16.79 ± 4.82 cm^2^, *p* < 0.001) and right ventricular internal diameter (RVID, from 4.13 ± 0.79 to 3.68 ± 0.59 cm, *p* = 0.001) at the 3‐month interval post‐BPA. These improvements were sustained or even enhanced by the 6‐month mark, with RAA and RVID further diminishing (to 14.46 ± 3.78 cm^2^ and 3.29 ± 0.54 cm, respectively; both *p* < 0.01). The TAPSE/PASP ratios showed progressive improvement from baseline (0.32 ± 0.13) to 3 months (0.42 ± 0.13) and continued to improve at 6 months following BPA (0.50 ± 0.11, *p* = 0.04 compared to 3 months post‐BPA).

**Conclusion:**

BPA has significantly ameliorated clinical conditions, hemodynamic profiles, and initiated a continued reversal in right heart remodeling in patients with CTEPH.

## 1 Introduction

Chronic thromboembolic pulmonary hypertension (CTEPH) is defined as group 4 pulmonary hypertension (PH), and is usually caused by pulmonary thromboembolism, which is characterized by progressive pulmonary vascular remodeling, vascular stenosis or occlusion, increased pulmonary artery pressure and pulmonary vascular resistance (PVR) as well as right ventricular (RV) overload [1]. These pathological processes can ultimately lead to right heart failure and death. Pulmonary endarterectomy (PEA) is a well‐established treatment for CTEPH with low in‐hospital mortality. However, nearly one‐third of CTEPH patients present residual PH following PEA, and approximately 40% of patients are not eligible for PEA due to the presence of peripheral chronic thrombus or other complications [2, 3].

Recently, balloon pulmonary angioplasty (BPA) has been developed as a treatment for CTEPH [4, 5]. BPA is an intervention that uses a balloon catheter to expand the stenosis of the pulmonary artery and improve blood supply. Currently, BPA is usually applied to patients who are not eligible for PEA, present peripheral‐type of CTEPH, encounter persistent and recurrent PH after PEA, or refuse PEA [6]. The efficacy and safety of BPA have been demonstrated by several studies [7, 8, 9]. In one study involving 26 patients with CTEPH, BPA resulted in a significant improvement in parameters of RV function, as assessed by echocardiography [10]. Analysis of data from the Database of Pulmonary Hypertension in the Polish Population also revealed that in 156 patients after final BPA, the mean pulmonary arterial pressure (PAP) and PVR were significantly decreased, and the measurements in 6‐min walking test (6MWT) were significantly improved [11]. BPA‐related pulmonary injury were observed in 6.4% of all sessions [11]. One recent systematic review and meta‐analysis involving 14 studies with 725 patients revealed that BPA resulted in a significant reduction in mean PAP and PVR and an improvement in cardiac index and measurements in 6MWT [12]. Periprocedural mortality rate was 2.1% in these patients [12]. These findings suggest that BPA is a promising and safe therapy for patients with CTEPH who are ineligible for PEA.

BPA has also been introduced to China in recent years. Herein, we performed a single‐center, retrospective study to assess the dynamics of hemodynamic changes and right heart remodeling in CTEPH patients undergoing BPA treatment.

## 2 Study Material and Methods

### 2.1 Population and Study Design

The study included a cohort of 31 adult patients who were consecutively enrolled from January 2019 to January 2021. Each patient was diagnosed with CTEPH and underwent BPA procedures at Shanghai Pulmonary Hospital. The criteria for inclusion in the study were: (1) an age range of 18–80 years; (2) a confirmed diagnosis of CTEPH following the 2015 ESC guidelines on the diagnosis and treatment of pulmonary arterial hypertension (PAH) [1]; and (3) patients unsuitable or declining PEA, who had completed BPA treatment successfully. Exclusion criteria specified: (1) individuals exceeding 80 years of age or younger than 18 years; (2) patients lost to follow‐up post‐BPA treatment; and (3) the presence of left heart disease or other pulmonary conditions that could cause PH, which was ruled out through diagnostic tests including computed tomography (CT), right heart catheterization (RHC), and two‐dimensional echocardiography.

### 2.2 Data Collection

Clinical data were collected from patients who met the specified inclusion and exclusion criteria at three time intervals: before BPA, and at 3 months and 6 months following BPA treatment. The data set included age, sex, World Health Organization functional class (WHO‐FC), levels of N‐terminal pro‐brain natriuretic peptide (NT‐proBNP), and echocardiographic parameters, all of which were extracted from the patients' medical records. RHC was conducted pre‐BPA and again at the 6‐month post‐BPA mark.

#### 2.2.1 BPA Procedure

Pulmonary artery interventions were conducted by two experienced interventionists using a streamlined approach [13]. Initially, a 7F vascular sheath (Cook Company, USA) was placed into the femoral vein, with heparin (50 U/kg) administrated thereafter. Hemodynamic parameters were assessed following this. To perform selective pulmonary angiography, which is a method pivotal for targeting vessels, with a 6F JR4.0 guided catheter (Cordis Company, USA) to the site. Guide wires, either the 0.014 inches Pilot 50 (Johnson & Johnson, USA) or Sion blue (ASAHI, Japan), were used to navigate through the vessels for the placement of a 2.0 × 16 mm balloon dilation catheter (Atlas Company, USA). Graduated upsizing of the balloon dilation was subsequently employed, including the use of larger 8–10 mm diameter over‐the‐wire balloons. Dilation was executed with 8–14 atmospheric pressure, lasting 5–10 s. The procedure was halted if fluoroscopy exceeded 30 min or contrast medium usage surpassed 150 mL, with the subsequent BPA planned for 4 weeks later.

#### 2.2.2 RHC

All patients in the study cohort underwent RHC using a Swan‐Ganz catheter (either 7‐Fr or 7.5‐Fr; Edwards Lifesciences LLC, Irvine, CA, USA). The hemodynamic assessment was comprehensive, encompassing the mean right atrial pressure (RAP), mean pulmonary artery pressure (mPAP), pulmonary artery wedge pressure, and venous oxygen saturation (SvO_2_). Cardiac output (CO) was determined through the thermodilution method using ice‐cold isotonic sodium chloride solution, performed as a set of three measurements for accuracy. The cardiac index (CI) is calculated based on CO and body surface area. PVR was calculated utilizing the accepted standard formula. RHC procedures were meticulously executed by experienced physicians in accordance with established standard operating protocols [1].

#### 2.2.3 Echocardiography

Echocardiography was performed using the GE‐VIVID 7 Dimension ultrasound system (GE, Vingmed, Horten, Norway). All echocardiographic measurements were performed according to the American Society of Echocardiography recommendations [14]. The following parameters were meticulously measured: right atrial area (RAA), right ventricular internal diameter (RVID), tricuspid annular plane systolic excursion (TAPSE), systolic pulmonary artery pressure (PASP), left ventricular end‐diastolic diameter (LVEDD), left ventricular ejection fraction (LVEF), left ventricular eccentricity index (EI), and the presence of pericardial effusion (PE). Tricuspid regurgitation (TR) was scrutinized from multiple views, including the apical four‐chamber, parasternal short‐axis at the aortic valve level, and the RV inflow tract views.

### 2.3 Statistical Analysis

All data was presented as mean ± standard deviation, median (interquartile range [IQR]), or percentage depending on data distribution. Comparisons of changes in hemodynamic and RV functional parameters before and after BPA were performed using paired *t*‐test. A *p* value of 0.05 was considered statistically significant. All statistical analyses were performed using SPSS Statistics for Windows (IBM, Version 22, IBM Corp., Armonk, NY).

### 2.4 Ethics Statement

This study complied with the Declaration of Helsinki and was approved by the ethics committee of Shanghai Pulmonary Hospital (Approval ID: L20‐223).

## 3 RESULTS

### 3.1 Baseline Characteristics of Participants

Clinical characteristics of patients enrolled (*n* = 31) at baseline are detailed in Table 1. In brief, the average age of patients was 63 ± 8 years, and the majority of patients were female (77.42%, 24/31). The majority of patients were graded as WHO‐FC Class III (80.65%, 25/31).

**Table 1:** Baseline characteristics of patients.

Clinical characteristics (*N* = 31)	Value
Age (years)	63 ± 8
Sex	
Male	24 (77.42%)
Female	7 (22.58%)
WHO FC	
II	6 (19.35%)
III	25 (80.75%)
Heart rate (beats/min)	73 ± 11
Serum Biomarker	
Serum creatinine (μmol/L)	69.87 ± 17.4
NT pro‐BNP (pg/mL)	614.6 (151.5–1607)
PaO_2_ (mmHg)	61.83 ± 13.02
PaCO_2_ (mmHg)	34.22 ± 4.57
Hemodynamic	
Mean PAP (mmHg)	45.53 ± 11.19
CI (L/min/m^2^)	2.79 ± 0.80
PVR (Wood unit)	8.33 (6.64–11.14)

*Note:* Values are *n* (%), mean ± standard deviation or median with interquartile range.

Abbreviations: BPA, balloon pulmonary angioplasty; CI, cardiac index; NT pro‐BNP, N‐terminal‐pro‐B‐type natriuretic peptide; PAP, pulmonary atrial pressure; PASP, pulmonary arterial systolic pressure; PVR, pulmonary vascular resistance; WHO‐FC, World Health Organization functional classification.

Among the 31 patients, 29 had inoperable lesions located in the pulmonary artery segment or subsegmental. The remaining two patients had more proximal lesions. Due to their advanced age and comorbidities, and following multidisciplinary discussions, they were deemed unsuitable for PEA for CTEPH. The types of lesions addressed with BPA include ring‐like stenosis lesions, web lesions, subtotal occlusion lesions, total occlusion lesions, and tortuous lesions.

Before BPA, RHC disclosed a mean mPAP of 45.53 ± 11.19 mmHg and a PVR with a median of 8.33 (IQR: 6.64–1.14) Wood units, coupled with a median NT‐proBNP level of 614.6 pg/mL, marking a considerable hemodynamic compromise. These baseline metrics underscored a critical health status among the study cohort.

All patients were on long‐term anticoagulant treatment. Specifically, 16 patients were taking warfarin, while 15 patients were using direct oral anticoagulants. Additionally, 30 patients were receiving targeted therapy for PH. Among them, 16 patients were on riociguat, 4 patients were using sildenafil, 7 patients were on an endothelin receptor antagonist (ERA), and 3 patients were receiving a combination of riociguat and ERA. During the BPA treatment phase, the medication regimens prescribed to patients were rigorously adhered to with no modifications.

### 3.2 Changes in WHO‐FC and NT‐proBNP Levels After BPA Treatment

**Figure 1:**
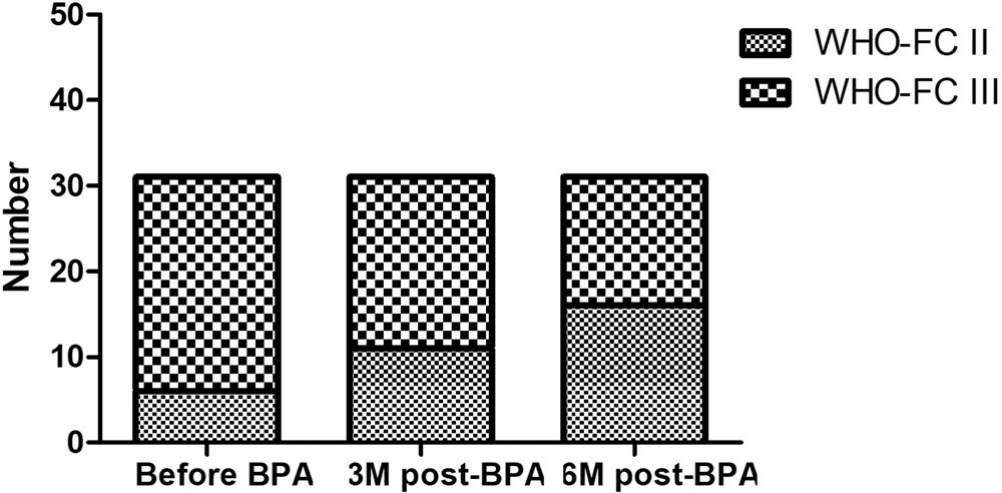
Changes in WHO‐FC before and after BPA treatment. Demonstrates a significant increase in the proportion of patients categorized as WHO‐FC II following BPA.

After 3 months post‐BPA treatment, the proportion of patients classified as WHO‐FC II increased from 19.35% to 35.48%. Furthermore, at 6 months posttreatment, the proportion of patients with WHO‐FC II increased to 51.61% (Figure 1). Additionally, NT‐proBNP levels exhibited a notable decrease from 614.6 pg/mL (IQR, from 151.5 to 1607) to 99.7 pg/mL (IQR from 40.4 to 349.1), *p* = 0.001. There was also a significant improvement in NT‐proBNP levels at 6 months post‐BPA 69.9 (44.7, 170.9) pg/mL compared to pre‐BPA (*p* < 0.001).

### 3.3 Hemodynamic Improvement Post‐BPA

**Figure 2:**
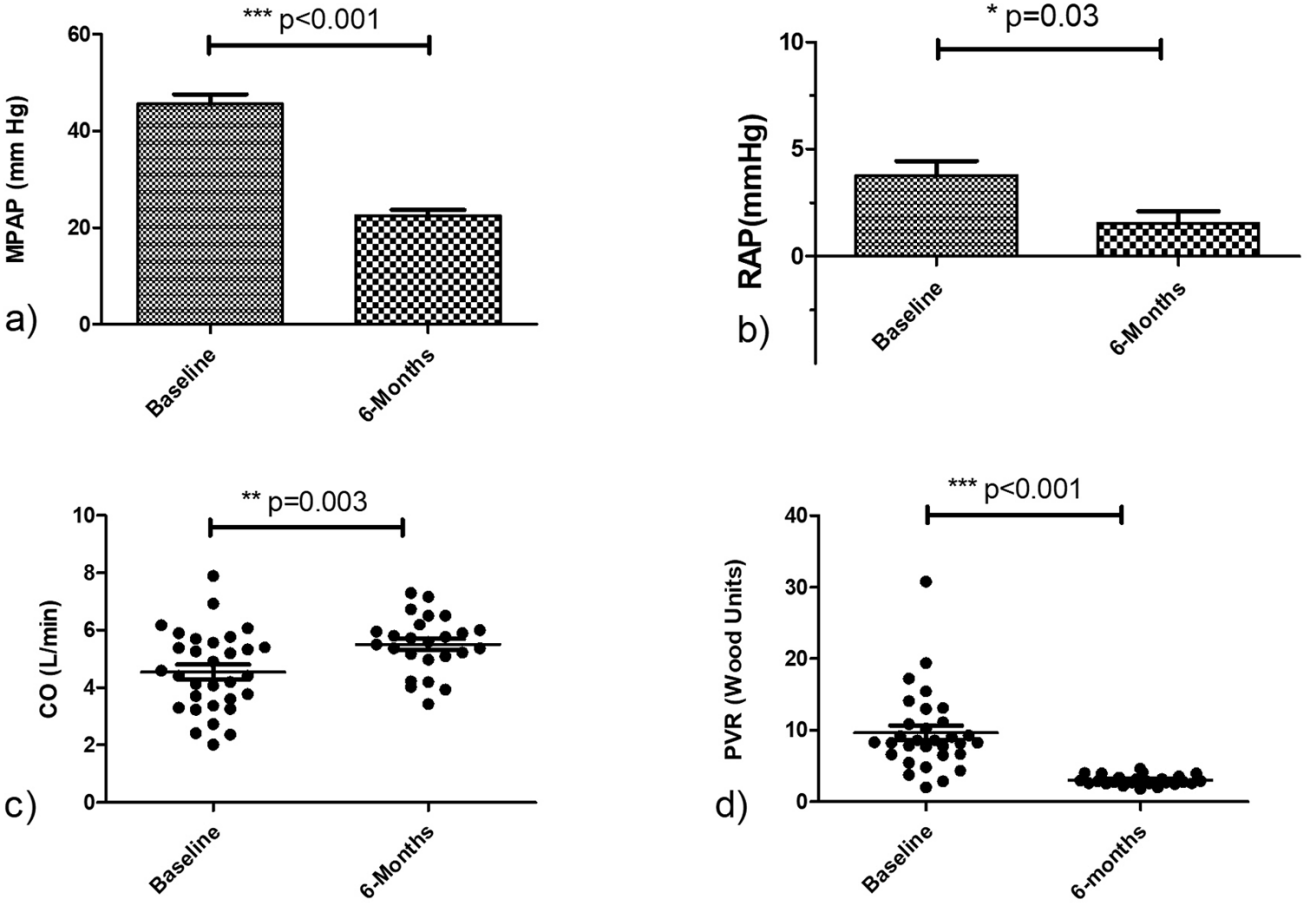
Hemodynamic enhancements observed 6 months after balloon pulmonary angioplasty (BPA) treatment. (a) MPAP significantly reduced from baseline. (b) RAP shows notable decrease from baseline. (c) CO notably increased relative to baseline. (d) PVR significantly lowered compared to baseline. CO, cardiac output; MPAP, mean pulmonary artery pressure; PVR, pulmonary vascular resistance; RAP, right atrial pressure.

Following the completion of BPA treatment at the 6‐month mark, there was a significant reduction in the mPAP from pretreatment levels (45.53 ± 11.19 vs. 22.56 ± 5.81 mmHg, *p* < 0.001). The mean RAP also experienced a notable decrease (3 [IQR: 2–5] vs. 1 [IQR: 0–2.5] mmHg, *p* = 0.03). In addition, there was a considerable decline in PVR when compared to initial values (8.33 [IQR:6.64–11.14] vs. 2.86 [IQR:2.60–3.47] WU, *p* < 0.001). Furthermore, there was a significant improvement in the CI from before BPA to 6 months after the procedure (2.75 ± 0.79 vs. 3.37 ± 0.49 L/min/m², *p* = 0.004) (Figure 2 and Table 2).

**Table 2:** Right heart catheterization parameters before and 6 months after balloon pulmonary angioplasty (BPA).

RHC parameters	Before BPA	6‐months post‐BPA	*p* value
Mean RAP (mmHg)	3 (2, 5)	1 (0, 2.5)	0.03
Mean PAP (mmHg)	45.53 ± 11.19	22.56 ± 5.81	< 0.001
Mean PAWP (mmHg）	6.81 ± 3.06	6.08 ± 1.00	0.44
CO (L/min)	4.47 ± 1.31	5.55 ± 1.00	0.003
CI (L/min/m²)	2.75 ± 0.79	3.37 ± 0.49	0.004
PVR (Wood unit)	8.33 (6.64, 11.14)	2.86 (2.60, 3.47)	< 0.001

*Note:* Values are *n* (%), mean ± standard deviation or median with interquartile range.

Abbreviations: CI, cardiac index; CO, cardiac output; PAP, pulmonary atrial pressure; PAWP, pulmonary artery wedge pressure; PVR, pulmonary vascular resistance; RHC, right cardiac catheterization.

### 3.4 Improvement in Right Heart Structure and Function After BPA Treatment

Three months after completing BPA treatment, a discernible enhancement was observed in both the structure and function of the right heart. The RAA significantly decreased compared to pre‐BPA (16.79 ± 4.82 vs. 20.29 ± 7.55 cm^2^, *p* < 0.001), the RVID showed a significant reduction compared to pre‐BPA (3.68 ± 0.59 vs. 4.13 ± 0.79 mm, *p* < 0.001), and TAPSE/PASP also significantly improved compared to pre‐BPA (0.42 ± 0.13 vs. 0.32 ± 0.13 mm/mmHg, *p* = 0.003). There were improvement in the EI (1.21 ± 0.14 vs. 1.32 ± 0.18, *p* = 0.081) and LVEDD (4.58 ± 0.40 vs. 4.29 ± 0.61 cm, *p* = 0.006) as well, although the improvement in EI did not reach statistical significance. Notably, the prevalence of moderate to severe TR was significantly reduced (38.7% vs. 12.9%, *p* = 0.04), as well as the incidence of PE (19.34% vs. 0%, *p* = 0.02). These findings are detailed in Table 3.

**Table 3:** Echocardiography parameters before versus 3 months and 6 months after balloon pulmonary angioplasty (BPA).

Parameters	Before BPA	3‐months post‐BPA	6‐months post‐BPA	*p* value (3 M vs. baseline)	*p value* (6 M vs. baseline)	*p* value (6 M vs. 3 M)
*Echocardiography*						
RA area (cm²)	20.29 ± 7.55	16.79 ± 4.82	14.46 ± 3.78	< 0.001	< 0.001	0.003
RVID (cm)	4.13 ± 0.79	3.68 ± 0.59	3.29 ± 0.54	0.001	< 0.001	0.001
PASP (mmHg)	68.07 ± 21.99	52.62 ± 17.25	43.22 ± 14.77	0.001	< 0.001	0.001
TAPSE (mm)	18.92 ± 2.61	20.30 ± 3.00	20.68 ± 3.11	0.03	0.02	0.54
TAPSE/PASP (mm/mmHg)	0.32 ± 0.13	0.42 ± 0.13	0.50 ± 0.11	0.003	0.036	0.04
EI	1.32 ± 0.18	1.21 ± 0.14	1.13 ± 0.07	0.08	0.023	0.35
LVEDD (cm)	4.29 ± 0.61	4.58 ± 0.40	4.57 ± 0.58	0.006	0.036	0.92
TR severity						
None	2 (6.45)	1 (3.23)	6 (19.34)	0.04	0.005	0.67
Mild	17 (54.84)	26 (83.87)	23 (74.19)			
Moderate	8 (25.80)	3 (9.68)	1 (3.23)			
Severe	4 (12.90)	1 (3.23)	1 (3.23)			
PE	6 (19.35)	0(0)	1 (3.23)	0.02	0.10	> 0.99

*Note:* Values are *n* (%), mean ± standard deviation or median with interquartile range.

Abbreviations: EI, eccentricity index; LVEDD, left ventricular end‐diastolic diameter; PASP, pulmonary arterial systolic pressure; PE, pericardial effusion; RA area, right atrial area; RVID, right ventricular internal diameter; TAPSE, tricuspid annular plane systolic excursion; TR, tricuspid regurgitation.

**Figure 3:**
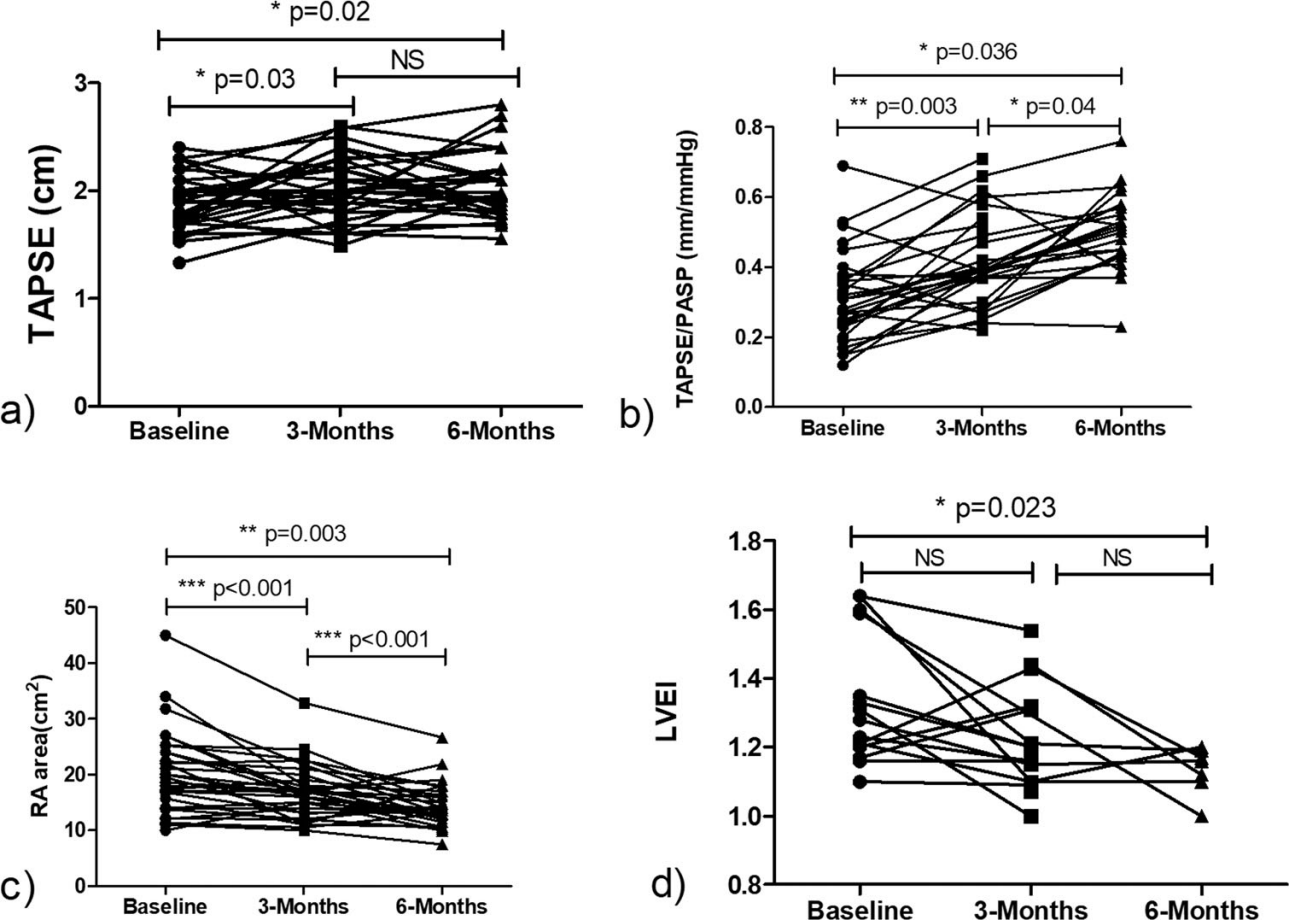
Sequential echocardiographic analysis of right ventricular remodeling and hemodynamics pre‐ and post‐BPA. (a) Significant TAPSE improvement at 3 months post‐BPA, maintained through 6 months. (b) Sequential improvement in TAPSE/PASP ratios after BPA shows marked enhancement from baseline at 3 months post‐BPA, with further improvement observed by 6 months. (c) Sequential improvement in RAA after BPA shows marked enhancement from baseline at 3 months post‐BPA, with further improvement observed by 6 months. (d) LVEI improvement observed 6 months following BPA from baseline levels. LVEI, left ventricular eccentricity index; PASP, pulmonary arterial systolic pressure; RAA, right atrial area; TAPSE, tricuspid annular plane systolic excursion.

Furthermore, during the follow‐up 6 months after the completion of BPA treatment, it was noted that the improvements in the structure and function of the right heart not only persisted but also exhibited continued enhancement when compared to the assessments made at the 3‐month post‐BPA mark. The RAA demonstrated a substantial decrease when compared to its status 3 months post‐BPA (14.46 ± 3.78 vs. 16.79 ± 4.82 cm^2^, *p* = 0.003). In addition to this marked reduction, there was a significant improvement in the TAPSE/PASP compared to the measurements taken at 3 months post‐BPA (0.50 ± 0.11 vs. 0.42 ± 0.13 mm/mmHg, *p* = 0.04), as illustrated in Figure 3.

Among the 31 patients, 4 cases experienced hemoptysis during the procedure, all classified as mild (< 50 mL/episode), which improved following balloon compression. Additionally, no patients experienced pulmonary artery wall dissection, reperfusion pulmonary edema, or hematomas.

## 4 DISCUSSION

This study provides compelling evidence that BPA offers significant benefits to patients with CTEPH, who are ineligible for PEA. The significant clinical improvements in WHO Functional Class, NT‐proBNP levels, RAA, RVID, and hemodynamic parameters such as mPAP and PVR observed at 3 and 6 months post‐BPA, underscore the efficacy of BPA in ameliorating disease severity and reversed RV remodeling for CTEPH patients.

Recent advancements have established BPA as a key component of routine care for CTEPH patients globally, indicated by over 1700 procedures reported in literature spanning Asia, the United States, and Europe [13, 15]. BPA has been effective in markedly reducing RV afterload, with substantial decreases in PVR and mPAP observed across various regions. European and Japanese data highlight significant improvements in hemodynamic parameters, reinforcing BPA's positive impact on patient outcomes [16, 17, 18]. Our study demonstrated significant hemodynamic improvements following BPA treatment in patients with CTEPH. Significant reductions were observed in mPAP and PVR, alongside an improvement in the CI, following BPA treatment, showcasing the procedure's efficacy in enhancing key hemodynamic parameters. These improvements align with international reports, highlighting the effectiveness of BPA in enhancing hemodynamic parameters [12, 19]. Noteworthy posttreatment observations included significant enhancements in cardiac function and reductions in NT‐proBNP levels, indicating an overall positive effect on cardiac function after reducing the right heart afterload. Whereas most international literature documents hemodynamic improvements immediately or within 24 h post‐BPA [19] or within a short term of 2–4 months [16], our assessment took place 6 months following the completion of all BPA procedures. This midterm evaluation more accurately represents the therapy's efficacy, further validating BPA's effectiveness.

As RV afterload decreases, an expanded right ventricle can undergo reverse remodeling. The extent and duration of the recovery of right heart structure and function post‐BPA are critical clinical concerns. Although MRI offers superior image clarity and volumetric accuracy, its use in assessing hemodynamics is clinically limited [20]. Echocardiography continues to be the cornerstone for evaluating cardiac function in CTEPH patients, effectively bridging the gap in understanding the dynamic changes in heart structure and performance after BPA.

Three months after completing BPA, our study observed notable improvements in PASP, RAA, RV diameter, and systolic function (TAPSE), along with the RV‐pulmonary artery coupling index (TAPSE/PASP), together with an increase in left ventricular diastolic diameter. Intriguingly, at the 6‐month mark post‐BPA, despite no further improvement in RV systolic function TAPSE being noted since the 3‐month evaluation, ongoing structural enhancements in the right heart were documented. This indicates that as afterload decreases, the RV's reverse remodeling process persists. Such sustained and progressive improvement underscores the long‐lasting beneficial effects of the BPA procedure on the right side of the heart over time.

In a study by Chen et al. [19] focusing on inoperable CTEPH patients, echocardiography performed within 24 h after BPA displayed considerable short‐term improvements in structural parameters, including right atrium diameter and RV end‐diastolic and systolic areas. However, functional parameters such as TAPSE and RV fractional area change showed no significant improvement in this immediate timeframe. This indicates that while BPA effectively reduces RV volume load and pulmonary pressure shortly after the procedure, enhancements in RV systolic function may not be immediate. Several studies highlight the efficacy of BPA in inducing reverse remodeling of the right heart in patients with CTEPH. Broch et al. [10] observed significant improvements in RV function and morphology, evidenced by increases in fractional area change and TAPSE and decreases in RV free wall peak strain, as well as reductions in RV dimensions and enhancements in left ventricular output following BPA in 26 CTEPH patients. Similarly, Tsugu et al. [21] reported that BPA led to significant enhancements in 3D RV volume, ejection fraction, and systolic peak strain, closely linked to hemodynamic improvements, including cardiac index and reduced RV dyssynchrony in 25 CTEPH patients, suggesting BPA's utility even in cases of mild hypertension. Kanar et al.'s research found that after BPA, despite no significant change in conventional echocardiographic measurements for CTEPH patients, speckle‐tracking echocardiography (STE) revealed substantial improvements. Specifically, the study noted a decrease in the electromechanical delay between the RV free wall and LV lateral wall and a reduction in the RV peak systolic strain dispersion index, confirming the effectiveness of BPA in enhancing RV mechanical synchronization and function in CTEPH patients [22]. 2D STE identifies RV free wall longitudinal strain as a key predictor of successful BPA outcomes in CTEPH patients [23]. Collectively, these studies underscore BPA's role in facilitating the physiological restructuring and functional recovery of the right heart in CTEPH, pointing towards its broader applicability in patient management and therapeutic outcomes.

Several limitations should be taken into account in the present study. Primarily, due to the retrospective, observational design conducted at a single center, the potential for selection bias is present and cannot be entirely excluded. Moreover, the cohort size was comparatively small, which could impact the generalizability of the results. Second, while the study provides clinical outcomes for patients at 3‐ and 6‐month intervals following BPA treatment, more extended follow‐up periods would be instrumental to thoroughly understanding the long‐term effects of BPA on hemodynamic changes, as well as RV remodeling and function. Future research is warranted to explore these dynamics more deeply.

In conclusion, the present study shows that BPA could be an effective therapy for patients with CTEPH who are not eligible for PEA surgery. BPA significantly improved hemodynamics as well as RV structure and functions and reversed RV remodeling. These improvements were sustained over time, as evidenced by follow‐up assessments at 3 and 6 months post‐BPA intervention.

## Author Contributions

Q.‐L.S., Q.‐H.Z., S.‐G.G., and J.‐M.L. spearheaded the study's conceptualization. Q.‐L.S., Q.‐H.Z., H.‐T.L., and J.D. crafted the methodology, while Q.‐L.S., Q.‐H.Z., J.H., and L.W. performed the statistical analysis. The initial draft was penned by Q.‐L.S. and Q.‐H.Z., with significant revisions and enhancements provided by S.‐G.G. and J.‐M.L. All authors have meticulously reviewed and approved the manuscript's final iteration.

## Conflicts of Interest

The authors declare no conflicts of interest.

## Data Availability

All authors have read and approved the final version of the manuscript. Jin‐Ming Liu had full access to all of the data in this study and took complete responsibility for the integrity of the data and the accuracy of the data analysis.
